# Programming of Renal Development and Chronic Disease in Adult Life

**DOI:** 10.3389/fphys.2020.00757

**Published:** 2020-07-16

**Authors:** Eugenie R. Lumbers, Yoga Kandasamy, Sarah J. Delforce, Amanda C. Boyce, Karen J. Gibson, Kirsty G. Pringle

**Affiliations:** ^1^School of Biomedical Sciences and Pharmacy, Faculty of Health and Medicine, The University of Newcastle, Callaghan, NSW, Australia; ^2^Hunter Medical Research Institute, Newcastle, NSW, Australia; ^3^Department of Neonatology, Townsville University Hospital, Douglas, QLD, Australia; ^4^School of Medical Sciences, University of New South Wales, Sydney, NSW, Australia

**Keywords:** renal, development, oligonephropathy, glomerular hypertension, renin, nutrition

## Abstract

Chronic kidney disease (CKD) can have an insidious onset because there is a gradual decline in nephron number throughout life. There may be no overt symptoms of renal dysfunction until about two thirds or more of the nephrons have been destroyed and glomerular filtration rate (GFR) falls to below 25% of normal (often in mid-late life) (Martinez-Maldonaldo et al., 1992). Once End Stage Renal Disease (ESRD) has been reached, survival depends on renal replacement therapy (RRT). CKD causes hypertension and cardiovascular disease; and hypertension causes CKD. Albuminuria is also a risk factor for cardiovascular disease. The age of onset of CKD is partly determined during fetal life. This review describes the mechanisms underlying the development of CKD in adult life that results from abnormal renal development caused by an adverse intrauterine environment. The basis of this form of CKD is thought to be mainly due to a reduction in the number of nephrons formed *in utero* which impacts on the age dependent decline in glomerular function. Factors that affect the risk of reduced nephron formation during intrauterine life are discussed and include maternal nutrition (malnutrition and obesity, micronutrients), smoking and alcohol, use of drugs that block the maternal renin-angiotensin system, glucocorticoid excess and maternal renal dysfunction and prematurity. Since CKD, hypertension and cardiovascular disease add to the disease burden in the community we recommend that kidney size at birth should be recorded using ultrasound and those individuals who are born premature or who have small kidneys at this time should be monitored regularly by determining GFR and albumin:creatinine clearance ratio. Furthermore, public health measures aimed at limiting the prevalence of obesity and diabetes mellitus as well as providing advice on limiting the amount of protein ingested during a single meal, because they are all associated with increased glomerular hyperfiltration and subsequent glomerulosclerosis would be beneficial.

## Introduction

Renal disease can have an insidious onset. Apart from congenital causes of chronic kidney disease (CKD) and syndromes that result in renal failure (Acute Kidney Injury, IgA nephropathy, glomerulonephritis, renal infections, and tumors), there may be no overt symptoms of renal dysfunction until about two thirds or more of the nephrons have been destroyed and glomerular filtration rate (GFR) falls to below 25% of normal (often in mid-late life) (Martinez-Maldonaldo et al., 1992). Once End Stage Renal Disease (ESRD) has been reached, survival depends on renal replacement therapy (RRT).

Chronic kidney disease causes hypertension and cardiovascular disease; and hypertension causes CKD. Albuminuria is also a risk factor for cardiovascular disease. Therefore, a stronger emphasis on assessing renal function by estimated glomerular filtration rate (eGFR) and albuminuria should be included in risk assessment tables for heart disease and hypertension, a conclusion drawn from a meta-analysis carried out in 2015 (Matsushita et al., 2015).

The early onset of CKD has its origins in fetal life. This review describes the mechanisms underlying development of CKD in adult life that result from abnormal renal development caused by an adverse intrauterine environment. The basis of this form of CKD is thought to be mainly due to a reduction in the number of nephrons formed *in utero*. However, as described below, renal programming of tubular function may also result in renal dysfunction. The effects of programming on renal development can also be sex specific (Gingery et al., 2009; Lankadeva et al., 2014).

Although the number of nephrons in adults varies widely (Hoy et al., 2003), it is useful to consider that each kidney has about 1,000,000 nephrons. Each nephron is composed of a large number of different cells, each with unique properties to carry out different functions. The correct assembly of these cells in terms of their location has to occur for normal renal function. It is for these reasons that neonephrogenesis cannot occur once nephrogenesis is complete, even though renal progenitor cells persist which are able to repair injured portions of existing nephrons (Romagnani et al., 2013, Figure 1). Since nephrogenesis in the human fetus is complete by term, no new nephrons can be formed after birth. Consequently, at this time and throughout childhood, the number of nephrons is greatly in excess of the number required to serve the metabolic mass of the individual until adult size is reached. A sufficient number and function of filtering nephrons underpins the successful regulation of fluid and electrolyte balance and blood pressure while rapidly eliminating unwanted toxins and by-products of metabolism. The kidney is the most complex structural organ in the body, so it is not surprising therefore that it is susceptible to alterations in its pattern of development.

**FIGURE 1:**
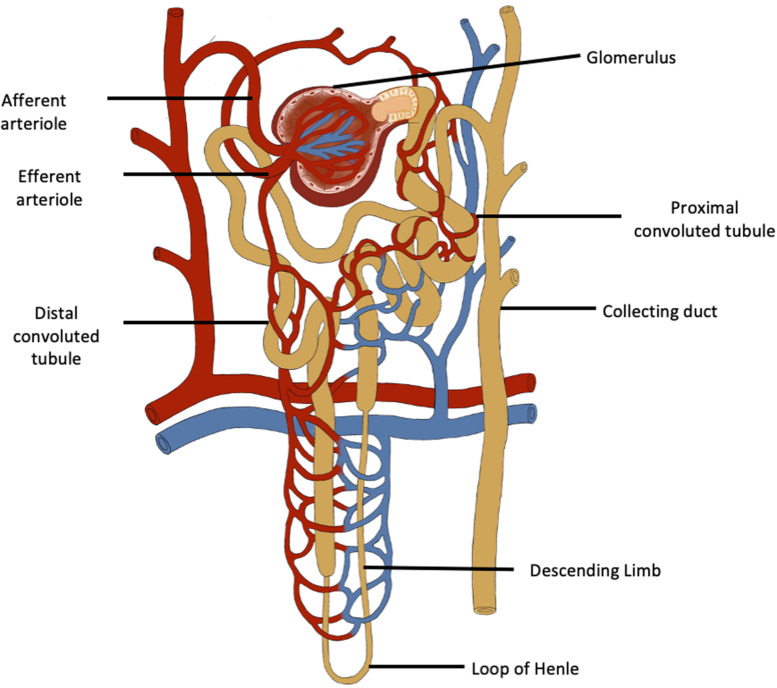
The functional unit of the kidney is the nephron which has a complex structure and a complex variety of cells. Neonephrogenesis cannot occur although there are renal progenitor cells, podocyte progenitor cells and tubular progenitor cells that can repair existing nephrons.

David Barker’s seminal research on the relationship between maternal health and programming of cardiovascular disease in offspring resulted in the development of a body of knowledge that exposed the potential long-term consequences of adverse intrauterine development on the predisposition to chronic disease in adult life, or the discipline now known as the Developmental Origins of Health and Disease (DOHaD) (Barker et al., 1989). This field of research examines the impact of the intrauterine and early life environment on offspring development and programming, particularly through epigenetic events. While it has been known for many years that ingestion by the mother of drugs like thalidomide (Taussig, 1962) or exposure to chemicals such as mercury, which caused Minimata disease (Matsumoto et al., 1965), were teratogens, there had been little appreciation that some chronic diseases that occur in adult life also have their origins *in utero*. Barker used birthweight as a measure of the degree to which maternal health had impacted on fetal development. He was the first to show that infants born with a low birth weight had a greater risk of developing hypertension, cardiovascular disease and diabetes mellitus in middle age (Zanetti et al., 2018).

Brenner and Chertow (1994) postulated that low birth weight infants also have fewer nephrons and this predisposes them, as adults, to systemic and glomerular hypertension as well as CKD. They based their hypothesis on three findings: (1) the association between birth weight and nephron number; (2) the inverse relationship between birth weight and hypertension in adult life; and (3) the inverse relationship between blood pressure and nephron number (Brenner and Chertow, 1994). These observations showed that abnormal renal development plays a pivotal role in the pathogenesis of the developmental origins of hypertension and cardiovascular disease.

The human fetus completes nephrogenesis before term [at 36 weeks of gestation (Osathanondh and Potter, 1963)]. Therefore, renal development in preterm infants born before 36 weeks is likely to be altered and may be affected by the adverse milieu of neonatal intensive care (e.g., hypotension, hypoxia and exposure to nephrotoxic antibiotics) (Keijzer-Veen et al., 2010; Sutherland et al., 2011). In females, the reduction in kidney volume effect persists into adult life (Keijzer-Veen et al., 2010).

In postnatal life an individual can be exposed to a variety of stressors that cause damage to nephrons. These include renal infections, diabetes mellitus and autoimmune diseases, e.g., streptococcal glomerulonephritis and IgA nephropathy. If an individual is born with fewer nephrons these conditions are more likely to cause an early onset of CKD compared with their impact on the renal health of individuals who are endowed with a full complement of nephrons at birth. This ‘second-hit’ therefore exacerbates the prevalence of renal disease (Hoy et al., 2006).

The question also arises as to whether maternal oligonephropathy can cause transgenerational changes in renal development and predispose her offspring to CKD, cardiovascular disease and hypertension in adult life. Does abnormal maternal renal function (occurring as a result of oligonephropathy) impact on fetal kidney development and renal function in her young offspring?

**FIGURE 2:**
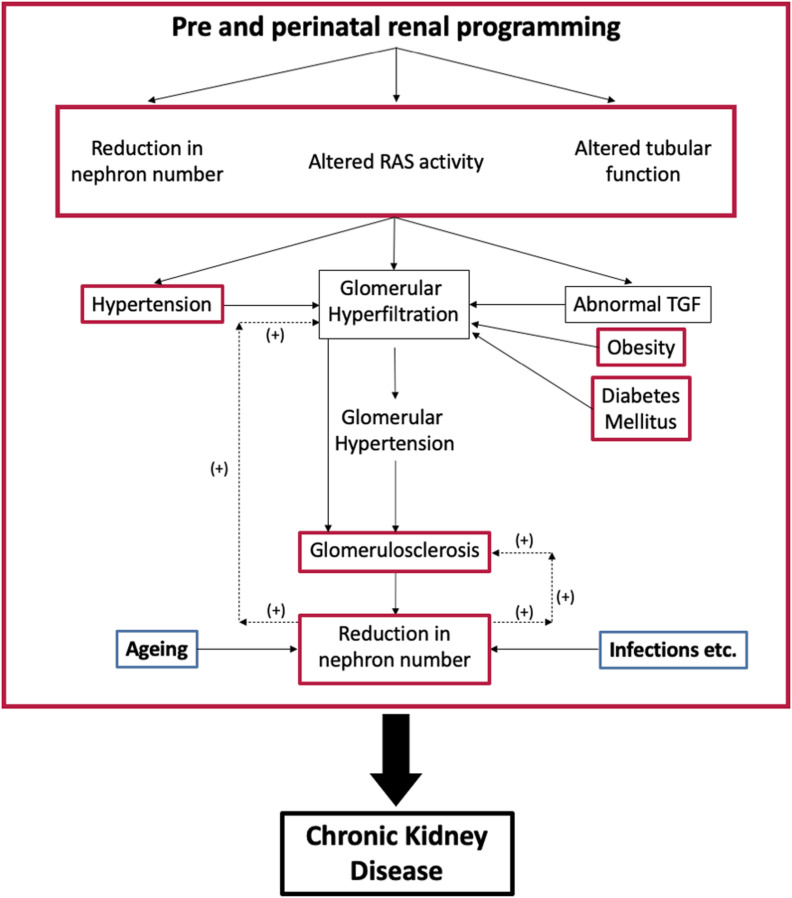
Interaction between programmed changes in renal development [oligonephropathy, tubular function and activity of the renin-angiotensin system (RAS); red] and postnatal health. Postnatal conditions like hypertension, diabetes mellitus, and obesity (red) cause glomerular hyperfiltration that can lead to glomerulosclerosis and a further reduction in nephron number. Aging and infection also affect nephron numbers (blue). TGF, tubuloglomerular feedback.

This review describes how an adverse intrauterine environment can alter nephrogenesis, cause oligonephropathy and possibly tubular dysfunction, so predisposing to CKD (Figure 2). It does not discuss the genetic defects that cause congenital abnormalities in the kidney and urinary tract (CAKUT). These are, however, relatively common accounting for about 40% of cases of ESRD in patients younger than 30 years (van der Ven et al., 2018).

**FIGURE 3:**
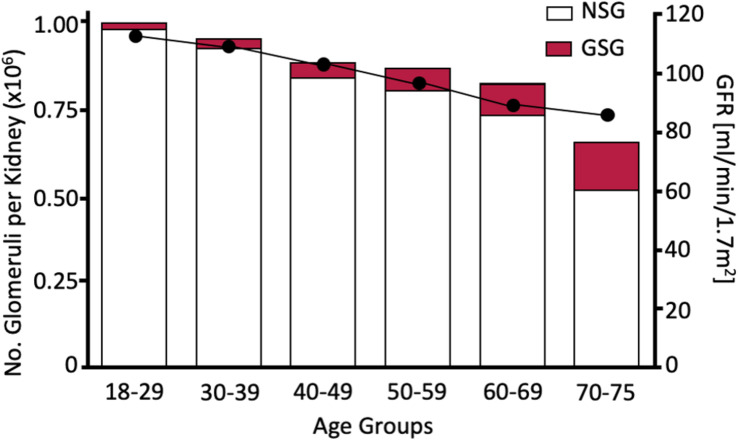
Age dependent increase in glomerulosclerotic nephrons (GSG) and decrease in non-sclerotic nephrons (left axis) and GFR. Since the total nephron population declines to <80% of the population measured in the 18–29 age group nephrons must have disappeared as well as becoming sclerotic. Redrawn from Denic et al. (2017).

Before addressing those factors that limit the intrauterine acquisition of nephrons, this review examines the physiological reasons behind why this predisposes individuals to an earlier onset of CKD in adult life, which is the necessity to maintain GFR regardless of the number of nephrons available to contribute to the formation of urine. It should be noted that throughout life there is a progressive loss of normal glomeruli and an increase in sclerotic glomeruli. It seems that glomeruli also disappear because the decrease in non-sclerotic glomeruli is not quantitatively matched to the increase in sclerotic glomeruli (Figure 3, Denic et al., 2017). Therefore, an individual who starts life with fewer nephrons will develop CKD at an earlier age than an individual endowed with a larger number of nephrons.

## The Conflict Between Glomerular Filtration Rate (GFR) and the Number of Nephrons Available to Filter the Blood

The first step in the formation of urine is the production of an ultrafiltrate of plasma generated by the pumping action of the heart, which forces fluid from the glomerular capillaries across the glomerular basement membrane (GBM) into the blind ending of the renal tubule known as Bowman’s capsule. The major force opposing the production of the plasma ultrafiltrate is the colloid osmotic pressure of the plasma proteins, which are not filtered. The GBM consists of endothelial cells, the glomerular membrane, and podocytes that wrap around the membrane. Podocytes have foot processes that are comprised of a functioning slit diaphragm in between a network of proteins. The outer surface of the foot processes have a negatively charged glycocalyx that faces into the urinary space and maintains the cytoarchitecture of the podocytes through charge repulsion. Thus this three layered barrier to filtration results in size and charge selective filtration (Reiser and Altintas, 2016). In the mammalian kidney the glomerular filtration rate (GFR) is high, with a GFR between 90 and 120 mL/min/1.73 m^2^ body surface area.

Metabolism is the sum of all the reactions occurring within the cells of the body. As a fundamental process it needs the cardiovascular system to supply sufficient nutrients to all metabolizing tissues and systems to efficiently eliminate the waste products of metabolism (lungs and kidneys). Each element within the system is interdependent and influenced by metabolic rate. Therefore, GFR, as the first step in the elimination of products of metabolism by the kidney, is set by the metabolic rate (Singer, 2001). In small animals like the shrew, which has a very high metabolic rate (oxygen consumption is 3–4 times higher than expected), the kidneys are 1.91% of the body weight (2.8 times greater than expected) (Singer, 2001).

By convention, GFR is normalized to body surface area (BSA) because metabolic rate is related to BSA and it was assumed that this would be more accurate as it eliminated changes in mass due to obesity. In adults of normal body weight, GFR/BSA and GFR not corrected for BSA are similar. In obese subjects, however, the calculation of GFR/BSA results in a value that is inappropriately low because BSA is calculated from height and weight. Table 1 compares the effects of indexing GFR (measured using [Cr^51^] EDTA) to BSA within female patients in a hospital nephrology unit who had low BMIs, with the effects of indexing GFR to BSA in females in the hospital metabolic disorders unit who had high BMIs. It can be seen that the difference between the mean absolute GFR and mean indexed GFR increases as BMI increases, i.e., the mean indexed GFR is inappropriately reduced because the greater weight of individuals used in the calculation of BSA. Since the number of nephrons does not increase after birth and they are not related to an individual’s fat mass, in obese subjects each individual nephron must form a greater volume of glomerular ultrafiltrate: this is masked by calculating eGFR. This possibly explains why obesity is a risk factor in patients with nephropathies (Geddes et al., 2008). In infants there are also a number of issues that complicate estimation of GFR indexed to BSA/1.73 m^2^ (Delanaye et al., 2005; Geddes et al., 2008).

**TABLE 1:** Mean difference between absolute (ml/min) and indexed (ml/min/1.73 m^2^) GFR and body mass index (BMI).

**BMI)**		**Mean absolute**	**Mean indexed)**	**Mean difference between**
**(kg/m2)**	***n***	**GFR**	**GFR (Dubois)**	**indexed and absolute GFR**
18.5–25	40	44.47	43.38	–1.09
>30	81	81.73	70.94	−18.2*
>40	33	110.17	87.76	−24.85*

*P < 0.0001 (Delanaye et al., 2005).

The overall absolute GFR is the result of filtration of plasma by about 1 million nephrons in each kidney. If the number of nephrons is reduced, GFR does not fall to a similar extent. For example, removal of 2/3 of the total renal mass (and therefore nephron number) in an animal model is associated with only a small 17% reduction in renal blood flow/g kidney weight and 16.5% reduction in GFR/g kidney weight. Since the number of nephrons was reduced to about 1/3 of the original population it follows that the remaining nephrons have to filter more (Gibson et al., 2006). This phenomenon is known as glomerular hyperfiltration. Glomerular hyperfiltration occurs in individuals with fewer than the normal complement of nephrons, for example after unilateral nephrectomy, especially early in life (Godron-Dubrasquet et al., 2017). Thus, individuals with a solitary kidney from early life onwards should have their renal function regularly monitored throughout life.

Occurring in ‘normal’ healthy individuals, glomerular hyperfiltration is said to be present if the GFR is greater than the 95% confidence limits for the patient’s age group. With aging, GFR declines so an apparently normal GFR in an elderly person can mask glomerular hyperfiltration. Hyperfiltration is often associated with albuminuria, possibly due to leakage across the glomerular barrier or failure of tubular protein reabsorption. Thus, albuminuria is a robust measure of renal dysfunction (Hoy and McDonald, 2004). In individuals with oligonephropathy, a so-called ‘normal estimated GFR’ (eGFR) (calculated from the serum creatinine) has to be the result of hyperfiltration.

Any form of proteinuria and glomerular hyperfiltration leads to secondary focal segmental glomerulosclerosis and the loss of nephrons (Rosenberg and Kopp, 2017). Glomerular hyperfiltration causes glomerular damage if there is raised intraglomerular hydrostatic pressure known as intraglomerular hypertension. This can be the result of hypertension, afferent arteriolar dilation or increased efferent arteriolar tone. Tubular sodium reabsorption also corrects GFR via tubuloglomerular feedback (TGF), so failure of TGF can cause glomerular hyperfiltration (Vallon, 2003).

In pregnancy, the increase in renal blood flow (RBF) is greater than the increase in GFR and there is no intraglomerular hypertension, so this physiological glomerular hyperfiltration probably does not result in long term renal damage (Helal et al., 2012). Furthermore, it is comparatively short-term. In conditions like obesity, where GFR increases to cope with increased metabolic demand, hypertension, and diabetes mellitus, there is intraglomerular hypertension (Helal et al., 2012).

## How Does Glomerular Hyperfiltration Damage the Nephron?

**FIGURE 4:**
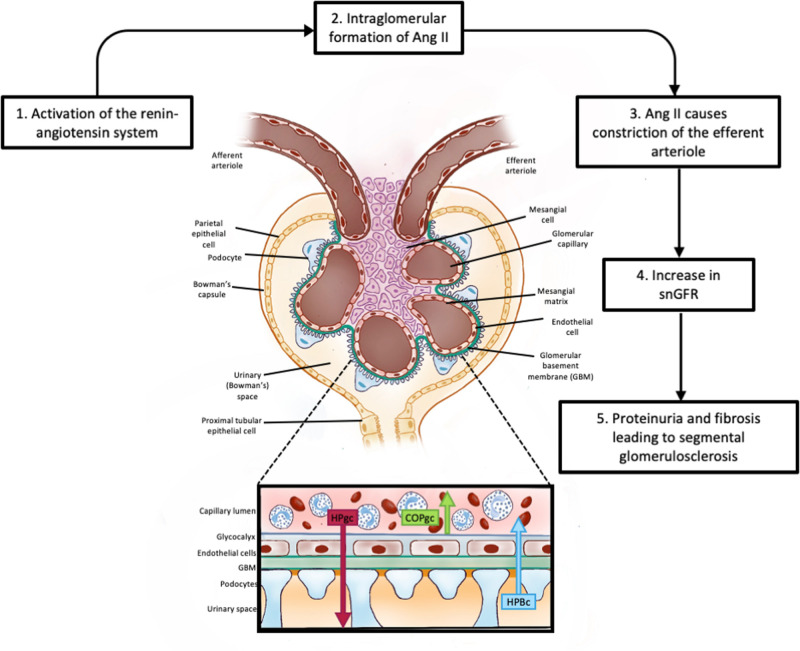
Glomerular filtration. This figure shows the basic cellular structure of the glomerulus; an enlargement of the filtration barrier (Kf) to ultrafiltration showing the two major forces involved in the formation of the glomerular ultrafiltrate (HPgc; the hydrostatic pressure within the glomerular capillaries). COPgc; the colloid osmotic pressure exerted by plasma proteins retained within the glomerular capillary. Other pressures affecting ultrafiltration include the hydrostatic pressure of the ultrafiltrate within Bowman’s capsule and the colloid osmotic pressure of any proteins that may have undergone ultrafiltration. Filtration equilibrium is reached when pressures favoring filtration are equal to pressures opposing filtration. The hydrostatic pressure within the glomerular capillary is influence by arterial pressure and by the degree of constriction of the efferent arteriole. Release of renin from the myoepitheliod cells lining the afferent arteriole generates angiotensin I which is converted to angiotensin II (Ang II) as it transits the glomerular capillaries. Ang II causes constriction of the efferent arteriole, raising HPgc and increasing filtration rate. SnGFR: is the single nephron glomerular filtration rate.

High intra-glomerular capillary pressures exert shear forces on the endothelial cells lining the capillary wall. The mechanical stresses caused by the consequent rise in filtration rate, cause the GBM to increase and therefore impose stress on the podocyte foot processes. Although the podocytes hypertrophy, mismatching of the increase in GBM area and the extent to which it is covered by podocytes can occur. The podocytes detach, leading to denuded areas of GBM. The result is segmental sclerosis. The high rate of filtration also causes mechanical stresses that dilate the urinary space and proximal tubules. In early diabetes mellitus the increase in sodium reabsorption is linked to increased glucose reabsorption with a reduction in tubuloglomerular feedback (TGF; Vallon, 2003) and release of inflammatory mediators that cause inflammation. The destruction of podocytes and glomerular inflammation leads to fibrosis and destruction of the glomerular capillary complex essential for filtration. This process is known as glomerulosclerosis. It is exacerbated when the pro-inflammatory arm of the renin-angiotensin system (RAS), the Angiotensin II/AT_1_R axis is activated either to maintain efferent arteriolar tone and hence GFR (Figure 4) as occurs in hypertension (Simons et al., 1994; Keller et al., 2003) or by activation of the renal G-coupled protein receptor, GRP91, by high glucose levels as occurs in diabetes mellitus (Peti-Peterdi, 2010). Alternatively, loss of the anti-inflammatory arm of the RAS [ACE2-Ang(1–7)-MasR], as occurs in COVID-19 infection, results in high levels of Ang II and loss of the anti-inflammatory peptide Ang-(1–7) causing acute renal failure (Cheng et al., 2020). Limiting these consequences of hyperfiltration by blocking the formation of Ang II or blocking its interaction with the AT_1_R are mainstays in the treatment of hypertension and diabetes.

Since nephrogenesis is complete by about 36 weeks of gestation, all the nephrons an individual will ever have are present at term. Thus, oligonephropathy caused by fetal growth restriction or in the early postnatal life of the preterm infant, may itself be associated with glomerular hyperfiltration (GH) because single nephron filtration rate (SNGFR) is increased. This can even occur *in utero* (Gibson et al., 2007; Turner et al., 2015). A high SNGFR at birth predisposes to an early onset of CKD. This is especially the case if the kidney is exposed to a second hit from those postnatal factors that also cause glomerular hyperfiltration (e.g., hypertension, diabetes mellitus, and obesity). The early onset of GH in individuals born with a fewer number of nephrons or who lose nephrons is, in early postnatal life undetected, as the eGFR will appear to be ‘normal.’ There is also strong evidence (Keller et al., 2003) that individuals with fewer nephrons are more likely to suffer from essential hypertension, which in turn causes glomerular hyperfiltration and glomerulosclerosis.

Intrauterine growth restriction is not only associated with a reduction in nephron number and hence CKD (Brenner and Chertow, 1994) but also with cardiovascular disease and diabetes mellitus. This triad or syndrome is inextricably linked through the adverse effects of poor renal function on cardiovascular health and the adverse effects of diabetes mellitus on renal health.

The long-term impact of pregnancy-induced hyperfiltration on the development of CKD is unknown. It can be assumed however, that conditions like hypertension, diabetes mellitus, and obesity occurring in pregnancy are likely to exacerbate any effects of pregnancy-induced hyperfiltration on glomerular and tubular integrity. Moreover, if the mother has oligonephropathy, any effects of pregnancy-induced hyperfiltration exacerbated by glomerular hypertension associated with obesity, the onset of hypertension, or gestational diabetes will predispose these women to an early onset of CKD.

Pregnancy induced hyperfiltration is not the only form of hyperfiltration occurring in response to physiological events. High protein meals/infusions of amino acids also cause hyperfiltration, probably as an evolutionary adaptation resulting in increased renal perfusion and glomerular filtration (Meyer et al., 1983a, b). Interestingly, glomerular hyperfiltration in response to amino acid infusions can be demonstrated in the fetus; it is associated with increased expression of angiotensinogen, the substrate for renin (Boyce et al., 2005). Human volunteers fed 50–70 g of protein had a 50% increase in GFR and patients maintained on total parenteral nutrition also had a 50% increase in GFR during the 12 h of amino acid infusion (Meyer et al., 1983b). The amount of protein in the diet influences renal health in remnant kidney animal models (Kleinknecht et al., 1979; Salusky et al., 1981). Thus, it can be assumed that when there is a severe reduction in nephron number at birth, a high dietary protein intake could hasten the rate of progression toward CKD in adult life.

## Development of the Kidney

Each nephron is composed of a large variety of cells, each with unique properties and functions. The correct assembly of these cells is required for normal renal function. The kidney is an extraordinarily complex organ. Briefly, three successive sets of kidneys develop during embryogenesis. These are the pronephros, the mesonephros and the metanephros; they are derived from the intermediate mesoderm. The pronephros begins to develop during the fourth week of gestation and deteriorates by the end of that week. The metanephros matures into the fully functional kidney. Only the metanephric mesenchyme can form renal tubules (Gilbert, 2000). The ureteric bud, which arises from the mesonephric duct (Wolffian duct), invades the mesoderm laterally. This epithelial mesenchymal interaction is driven by factors secreted by the condensing mesenchyme [notably *GDNF/c-RET/Wnt11* (Yosypiv, 2008) and hepatocyte growth factor (HGF)] and leads to budding and the formation of the collecting tubules, calyces, the renal pelvis and ureters.

Conversely, the ureteric buds stimulate differentiation of the adjacent mesoderm into the metanephric blastema, which forms the glomerulus, capsule and tubules of the nephron. The structural differentiation of the condensed mesenchyme around the ureteric vesicle is driven by the transcription factor (WT1) and a host of factors secreted by the ureteric bud including fibroblast growth factor 2 (FGF2), which maintains WT1 transcription, and bone morphogenetic protein (BMP7). Leukocyte Inhibitory factor (LIF) begins epithelial transformation of aggregated mesenchymal cells that is induced by FGF2 and stimulates secretion of Wnt4 (found in S-shaped tubules), which completes the transition to epithelial renal tubules. For a detailed and excellent description of nephrogenesis see Gilbert (2000). The S-shaped body, which forms the glomerular cleft (Bowmans capsule), is lined by podocyte progenitor cells while renal progenitor cells and tubular progenitor cells line the tubules. Capillaries invaginate the blind ending Bowman’s capsule and form the glomerular tuft. Further differentiation occurs as gestation continues.

Ureteric budding determines how many nephrons are formed. This process is known as branching morphogenesis as the ureteric bud divides into two buds, which each divide into two buds and so forth. The arterial tree undergoes a similar form of fractal branching directed by renin precursor cells. A new arteriolar branch is formed by the coalescence of renin cells around the site at which a new vessel will sprout. The elongating vessel is covered by renin containing cells which, like smooth muscle and juxtaglomerular cells, are driven to differentiate by the transcription factor, *Foxd1* (Gomez, 2017).

The formation of nephrons occurs radially so that the outermost nephrons are the most immature. This means that renal blood flow is lower in outer nephrons and the amount of oxyhaemoglobin reaching the outer cortical nephrons is lowest. In preterm infants, some outer glomeruli are often seriously damaged making it unlikely that they will ever filter plasma (Black et al., 2013).

## Abnormal Renal Development

**FIGURE 5:**
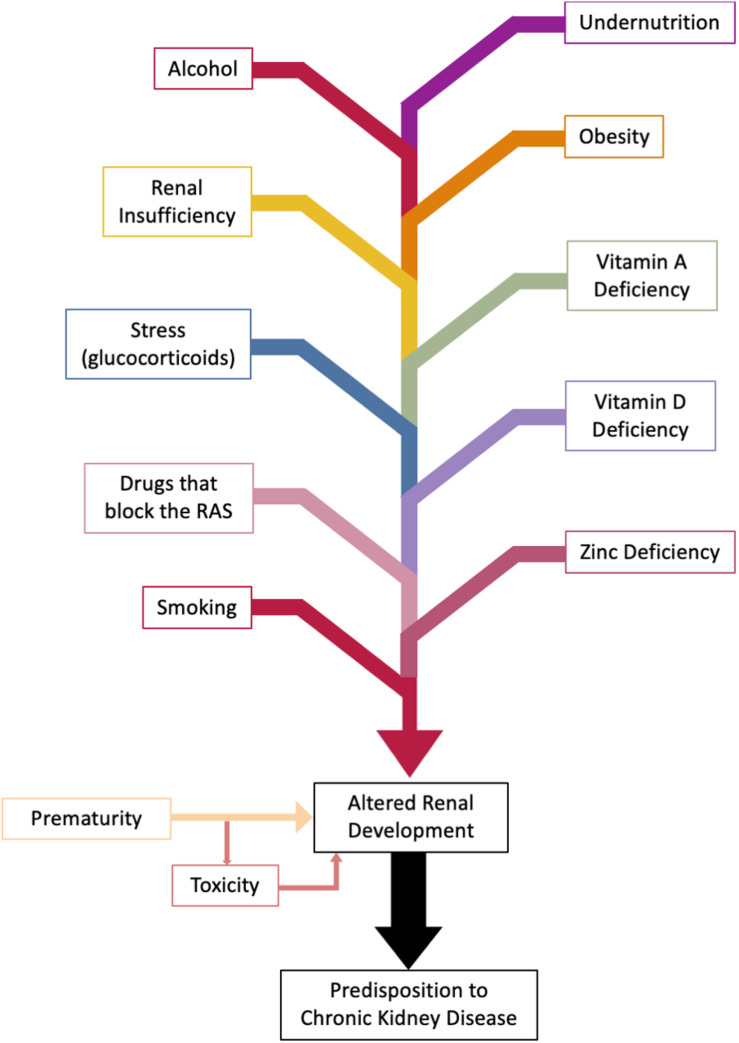
Factors altering fetal and perinatal renal development. Nephrogenesis is complete at 36 weeks of gestation. Effects on kidney development can be wide ranging from oligonephropathy, glomerular hypertrophy, tubular defects (Chen et al., 2004), and altered activity of the fetal renin-angiotensin system (RAS). Genetic factors are excluded.

Abnormal renal development can occur as a result of congenital anomalies, effects of the intrauterine environment, or as a result of prematurity (Figure 5).

### Genetic

The various genetic defects that are associated with CAKUT are not discussed further. Suffice to say that CAKUT represents 20–30% of all prenatally detected congenital anomalies (Queisser-Luft et al., 2002).

At a less clinically definable level Zhang et al. (2008) showed that a common polymorphism in the *RET* gene (*RET*^1476A^) was associated with about a 10% decrease in kidney volume and a 9% increase in Cystatin C (a measure of GFR) in newborns. Interestingly, the correlation between the hypomorphic *RET*^1476A^ with another hypomorphic SNP (*PAX*^*AAA*^) was associated with a 23% reduction in kidney volume compared with homozygotes carrying *RET*^1476G^ and *PAX*^*GGG*^. *RET* and *PAX* mutations act synergistically to affect branching morphogenesis. Zhang et al. (2008) showed that disrupting branching morphogenesis affects nephron number. Kidney volume and nephron number are strongly associated in the neonate (Zhang et al., 2008).

Some of the effects of blockade of the renin-angiotensin-aldosterone system (RAS) on renal development are similar to those seen in some congenital renal abnormalities. In particular, RAS blockade is associated with oligonephropathy and abnormal vascular development (Tufro-McReddie et al., 1995). Chen et al. (2004) have reviewed the effects of blockade of the RAS on renal tubular function. Furthermore, Angiotensin (Ang) II acting via the Ang II type 1 receptor (AT_1_R) stimulates ureteric bud branching by blocking *Spry 1*, releasing the *GDNF/c- RET/Wnt 11* pathway, the major stimulator of ureteric budding. Acting via the Ang II type 2 receptor (AT_2_R), Ang II upregulates *Pax2* and stimulates ureteric branching (Yosypiv, 2008).

The (pro)renin receptor (*ATP6AP2*, (P)RR) probably plays a role in renal development (Song and Yosypiv, 2011), however, deletion of the gene is lethal to the embryo, so it is more difficult to generate viable knockout models. Yosypiv et al. (2019) showed that while kidney stromal cell (P)RR was not necessary for ureteric branching, in conditional knockouts the lack of (P)RR affected arterial development and glomerular number. Since these knockouts had fewer renin containing cells the authors concluded that stromal (P)RR has an essential role in development of renin-expressing cells required for the proper development of the renal arterial tree. Abnormal expression of the renal RAS has been found in a number of animal models in which renal programming has been studied (Woods et al., 2001; Moritz et al., 2002).

### Environmental

The Barker hypothesis was based on epidemiological evidence showing that individuals who were small at birth were more likely to develop hypertension, cardiovascular disease and type II diabetes in later life. Events like the Dutch famine (Painter et al., 2005) and famine in China (Wang et al., 2018) also showed an association between malnutrition during pregnancy and birth weight. In both cases, low birth weight was associated with proteinuria or albuminuria, signs of renal dysfunction in adult life. Hinchliffe et al. (1992) also showed that nephron number was reduced in infants that were small at birth.

It is very difficult to measure nephron numbers in human populations because the kidneys have to be removed to count nephrons. Zhang et al. (2008) showed that in infants who died prior to 3 months of age, there was a strong correlation (*P* = 0.019) between kidney weight and the number of glomeruli. Regression analysis showed that there were 23,549 additional glomeruli/g kidney tissue (95% confidence interval of 4,590–42,238 glomeruli/g). A surrogate marker of kidney weight is the measurement of kidney volume using ultrasound. Ultrasound measurement of kidney volume before birth therefore provides a very useful surrogate measure of nephron number. Zhang et al. (2008) measured total renal volume by ultrasound and confirmed that there was a relationship between renal volume and glomerular number in a study that looked at the associations between common genetic variants and kidney size.

Since nephrogenesis is complete before birth, kidney volume at birth provides the best estimate of nephron number because postnatal renal growth is due to growth of the individual nephrons, in particular the renal tubules. There is a consensus that small for gestational age babies have smaller kidney volumes (Mishra et al., 2014). Therefore, it was logical that animal models, developed to study the developmental origins of chronic disease, first investigated the effects of reduced intrauterine growth due to undernutrition on nephrogenesis. Studies in animal models have shown that maternal health has a strong impact on the number of nephrons that her offspring develop (Figure 5). Specific factors that have been shown to be involved are:

(1) Maternal malnutrition/obesity;
(2) Maternal micronutrient deficiency (Vitamins A and D, zinc);
(3) Maternal smoking and alcohol intake;
(4) Administration of RAS blocking drugs;
(5) Maternal glucocorticoids; and
(6) Maternal renal dysfunction.

Some of these associations share a common pathway via which they influence fetal renal growth. For example, RAS blocking drugs (Lumbers et al., 1993; Barr, 1994; Woods et al., 2001; Lasaitiene et al., 2003), maternal undernutrition (Woods et al., 2001), Vitamin D deficiency (Boyce et al., 2013) or treatment with glucocorticoids in early gestation (Peers et al., 2001; Moritz et al., 2011), are all associated with altered activity of the RAS in the developing kidney and may exert their programming effects by altering early renal RAS expression or activity. On the other hand, oxidative stress and inflammation have been implicated in the etiology of oligonephropathy associated with maternal obesity, zinc deficiency and smoking (Tomat et al., 2011; Jackson et al., 2012; Flynn et al., 2013). Maternal alcohol and Vitamin A deficiency are associated with reduced expression of key genes involved in branching morphogenesis but oxidative stress may also play a key role (Gray et al., 2010). Furthermore, factors that restrict fetal growth are associated with smaller kidney volumes at birth and therefore are associated with oligonephropathy. As there is often catch-up growth after birth they will go through life with fewer nephrons and one can predict that they will when coupled with the age dependent decline in nephrons that occurs in healthy people develop CKD earlier than infants who appropriately grown for their gestational age (Kandasamy et al., 2014).

Prematurity also adversely affects kidney development because nephrogenesis is not complete at birth and therapeutic interventions can be toxic for the developing kidney.

#### Maternal Diet

##### Malnutrition

The effects of maternal global nutrient restriction (GNR) on the development of the kidneys using animal models has been extensively reviewed by Lee et al. (2019a). Lee et al. (2019a) concluded that maternal global nutrient restriction during pregnancy had a detrimental effect on kidney structure and function, that is, the kidneys were smaller, there was oligonephropathy and GFR was lower.

Pregnant rats fed a low-protein diet have small offspring. These animals have small kidneys and, in a number of studies, the offspring develop hypertension in adult life (Woods et al., 2001). They have also been shown to have fewer nephrons. Interestingly, the level of expression of renin in the kidneys of these animals is depressed. This reduced level of expression of renin, which is essential for both normal glomerulogenesis (Tufro-McReddie et al., 1995) and tubular development (Yosypiv, 2008) could be due to a deficiency in amino acids in the developing embryo, as a sustained supply of amino acids during intrauterine life stimulates the renal RAS (Boyce et al., 2005). Langley-Evans et al. (2003) cite data showing that supplementation of a low protein diet with glycine, alanine, or urea restored renal morphology and glomerular number but only glycine supplementation in the maternal diet prevented hypertension in offspring. This suggests that there is a dissociation between oligonephropathy and hypertension in rat offspring fed a low protein diet. This finding may be restricted to this model of oligonephropathy, as unilateral nephrectomy of the ovine fetus in mid-gestation is associated with hypertension at 6 months postnatal age (Singh et al., 2009). Sheep, like humans, complete nephrogenesis before birth.

##### Obesity

Although malnutrition is endemic in the developing world, in first world countries there is an epidemic of obesity. In animal studies a high-fat maternal diet (lard) resulted in offspring developing a syndrome similar to metabolic syndrome with obesity and hypertension. In these animals a high-fat diet did not affect kidney weight and glomerular number in offspring, but renal renin activity and renal Na-K-ATPase activity was suppressed (Armitage et al., 2005).

Studies by Jackson and Flyn clearly demonstrated that both pre- and postnatal exposure to a high fat/fructose diet caused changes in the kidney that were characteristic of long-standing inflammation and there was a synergistic effect of maternal obesity and a high-fat diet after birth (Jackson et al., 2012; Flynn et al., 2013). These changes (including an increase in glomerulosclerosis index), which were attributed to oxidative stress, were most severe in offspring exposed to a high-fat/fructose diet throughout the whole of gestation and in the early postnatal period (Jackson et al., 2012; Flynn et al., 2013). Glastras et al. (2016) also found that maternal obesity due to a high fat/fructose diet was associated with oxidative stress in offspring. They concluded, however, that postnatal exposure to this diet induced an overwhelming effect compared with diet-induced maternal obesity.

Lee et al. (2019b) found that fetuses of obese Indigenous women had smaller kidney volumes relative to their estimated body weights. This was because the fetuses were bigger than fetuses carried by Indigenous women with lower body weights. Since GFR is linked to metabolic rate and BSA it follows that these fetuses were born hyperfiltering. Coupled with their propensity to have higher body weights during childhood it is likely that in these children obesity will contribute to an increased risk of CKD in adult life (Lee et al., 2019b).

#### Micronutrients: Vitamins A, D and Zinc

##### Vitamin A deficiency

Vitamin A is essential for normal cell growth and development, so it is not surprising that severe Vitamin A deficiency leads to fetal death or congenital abnormalities (Wilson et al., 1953). Mild vitamin A deficiency is, however, relatively common (Merlet-Benichou et al., 1999). Lelievre-Pegorier et al. (1998) carried out an elegant study in pregnant rats who were exposed to vitamin A deficiency throughout gestation and showed that there was a strong correlation between the number of glomeruli in offspring and fetal retinol levels (*r* = 0.83, *P* < 0.001). Metanephroi from vitamin A deficient embryos grew less effectively *in vitro* but they could be rescued by supplementation with retinol (Lelievre-Pegorier et al., 1998). Retinol levels determined levels of expression of c-ret, a key pathway in nephrogenesis (see above). In an exciting study it was shown by Makrakis et al. (2007) that administration of retinoic acid to pregnant rats on a low protein diet offset the effects of protein deficiency on glomerular number.

Although these animal studies conclusively show a role for vitamin A deficiency in determining nephron number there are no definitive studies in human populations. Since mild vitamin A deficiency is common when there is poverty, these studies need to be carried out because mild vitamin A deficiency could account for smaller kidneys in these populations (Goodyer et al., 2007).

##### Vitamin D deficiency

A deficiency of vitamin D in pregnant women is quite common (Lips, 2010). Since the major source of vitamin D is from the action of ultraviolet light on the skin, it follows that the fetus can only obtain adequate levels of vitamin D from its mother. Formation of active vitamin D or 1,25 dihydroxycholecalciferol (1,25(OH)_2_D_3_) from 25(OH)D_3_ (the precursor to Vitamin D, which is formed in the liver from cholecalciferol produced in the skin) occurs in the kidney.

The effects of vitamin D on kidney development is confusing. On the one hand, vitamin D appears to increase glomerular development (Rogers et al., 2004), yet maternal vitamin D deficiency also increases nephron number (Maka et al., 2008; Nascimento et al., 2012). For these reasons the effects of both maternal Vit D deficiency and excess on offspring nephron endowment were studied and it was found that there were more glomeruli per milligram of kidney at PND20 in offspring of deficient mothers (78.09 ± 2.63 Nglom/mg) than in both replete and high vitamin D mothers (55.42 ± 2.83 and 58.26 ± 3.69, *P* = 0.001) (Burkitt et al., 2014). Although offspring nephron number was increased by maternal vitamin D deficiency, these additional nephrons could be abnormal. Boyce et al. (2013) found that adult male offspring of vitamin D deficient dams who ate normal chow from weaning had a reduced creatinine clearance and others have reported high blood pressure and ventricular hypertrophy in young offspring maintained on a vitamin D deficient diet (Gezmish et al., 2010; Tare et al., 2011).

Vitamin D acts via the vitamin D receptor (VDR), a nuclear receptor that regulates the transcription of a large number of genes. Since the VDR interacts with a cAMP response element (CRE) in the REN gene to suppress renin gene expression (Li et al., 2002), a deficiency of Vitamin D in the fetus could upregulate renin expression. Boyce et al. (2013) showed that offspring of pregnant rats subjected to a diet low in maternal vitamin D had increased renal renin expression at E20 (during nephrogenesis) and in adult life. This suggests that vitamin D deficiency could increase nephron number via RAS stimulation.

In contrast to the findings of Boyce et al. (2013) at E20 and adulthood, at PND20 it was subsequently found that several RAS genes, including renin, were downregulated (Burkitt et al., 2014). While downregulation at this stage (when nephrogenesis has been completed) does not negate the hypothesis that vitamin D deficiency stimulates nephrogenesis via upregulation of the RAS, it is does raise the possibility that there may be an alternative mechanism. In many tissues vitamin D inhibits cell proliferation and stimulates differentiation (Brown et al., 1999). Thus, vitamin D deficiency might stimulate proliferation and inhibit maturation of nephrons. Given that the increase in nephron number was associated with reduced renal function in adulthood, it is possible that the initial stages of nephrogenesis were stimulated but maturation was impaired resulting in poor renal function.

Notably, the effects of Vitamin D deficiency were sex specific. In both sexes during fetal life *REN* expression was increased and the kidney to body weight ratio was reduced at PND3 and PND20 but they were not different at 22 weeks. In males from Vit D deficient pregnancies, the renin levels were two times control levels. Adult males also had a reduced creatinine clearance, solute excretion and suppressed urinary Na/K ratio suggestive of increased aldosterone secretion. In females, the GFR was normal and the urine dilute because they drank more.

It is also interesting to note that even though glomerular number was increased in Vit D deficient offspring, kidney to body weight ratios were reduced despite the overall reduction in body weight, which further suggests abnormal tubular development (Boyce et al., 2013).

##### Zinc deficiency

Marginal or moderate zinc deficiency is common. Zinc plays an essential role in gene expression and organ development because it is present in over 300 enzymes where it plays a role in catalysis. Its levels affect the activity of NO producing enzymes (NOS synthase), antioxidants such as glutathione and enzymes involved in free radical scavenging such as glutathione peroxidase and catalase. As well zinc inhibits the activity of NADPH oxidases that catalyze the production of superoxide anion from oxygen. Free radicals activate inflammatory pathways leading to cell death (Tomat et al., 2011).

Therefore, it is not surprising that low maternal intake of zinc has marked effects on renal development and blood pressure in male offspring (Tomat et al., 2007). Male rats had oligonephropathy associated with a reduced GFR (Tomat et al., 2008), proteinuria and renal fibrosis, increased evidence of renal apoptosis and higher lipid peroxidation products. Catalase and glutathione peroxidase activities were reduced as were glutathione levels (Tomat et al., 2008). In male offspring of rats fed a low zinc diet and sacrificed at 6 days of age, the activity of the renal RAS was significantly altered; in particular the pro-inflammatory Ang II/AT_1_R axis was activated, ACE abundance was greater and the Ang II/ANG (1–7) ratio greater than in male rats fed a normal zinc diet (Gobetto et al., 2020). These effects were sex specific. Although both male and female zinc deficient rats had a greater abundance of AT_1_R, the Ang II/AT1R axis was not activated in female rats but Ang (1–7) levels were increased as was the abundance of the AT_2_R (Gobetto et al., 2020). Thus, the anti-inflammatory arms of the RAS were more active in females and probably protected them from the effects of the dysfunctional RAS and its associated inflammatory effects on oligonephropathy and renal artery modeling seen in male offspring.

#### Maternal Smoking and Alcohol

The adverse effects of smoking and alcohol on fetal growth, development and postnatal health are well known. Alcohol in high doses is a teratogen (Caputo et al., 2016), smoking reduces fetal growth (Diehm et al., 2017) but do either/both have specific effects on renal development that could contribute to the early onset of CKD? The answer is yes.

##### Smoking

Smoking during pregnancy leads to intrauterine growth restriction. We showed in an Indigenous cohort that smoking during pregnancy was associated with a reduction in birth weight of 327 g and smaller kidney volumes (Diehm et al., 2017); these infants therefore had oligonephropathy and were hyperfiltering from birth.

There were fewer nephrons as adults in the offspring of Balb/C mice exposed to cigarette smoke during pregnancy and lactation and the albumin/creatinine ratio was increased (Al-Odat et al., 2014). The renal expression of a pro-inflammatory marker, macrophage chemoattractant protein 1 (MCP1) was also increased. The authors point out that there are 4,000 chemicals in cigarette smoke that could impact on renal development. They showed an altered pattern of expression of key growth factors involved in nephrogenesis such as GDNF1, Pax 2 and Wnt II, all of which were increased at postnatal day (PND) 1 and a reduction in FGF 7 and 10. These patterns of expression were different at PND20 and similar to controls at week 13. It is suggested that oxidative stress plays a key role (Kabuto et al., 2004).

Taal et al. (2011) studied over 1,000 children from smoking and non-smoking mothers from 30 weeks of gestation to 2 years of age and concluded that consumption of >10 cigarettes/day was associated with a smaller kidney volume at this age.

##### Alcohol

Fetal alcohol syndrome (FAS) is best known for its effects on cognition, learning, and emotional and social development but it has widespread effects on growth and organogenesis (Caputo et al., 2016). The effects on the kidney are subtle. Moore et al. (1997) reported that light drinking (<3 glasses per week) was not associated with renal anomalies while moderate drinking (3–13 glasses per week) was associated with an increased risk of renal agenesis (odds ratio, 2.5; 95% confidence interval, 1.2–5.1).

Animal studies do, however, show a convincing effect of alcohol on renal development. In the sheep, a moderate to high intake [0.75 g/kg i.v. given daily from 95 to 133 days of gestation, maternal peak blood ethanol level (BEC) was 0.12g/dL] over the last third of gestation caused oligonephropathy [about 46,000 fewer nephrons (Gray et al., 2008)] and rats gavaged with 1 g/kg body weight ethanol on days 13.5 and 14.5 gestation had reduced nephron numbers and impaired branching morphogenesis (Gray et al., 2010). In both studies alcohol intake was high (BEC was 0.107 g/dl, 1 h after dosing rats). Alcohol caused a reduction in body weight so that kidney:body weight ratios appeared higher in female offspring of treated rats at PN30 than controls, but they were the same as controls in adult life. However, both male and female offspring of treated dams had 10–20% fewer nephrons. The relative expression of key genes involved in branching morphogenesis, GDNF, FGF7, Wnt11, TGFβ2, and TGFβ3 was reduced in E15.5 kidneys of ethanol-exposed fetuses (Gray et al., 2010). Metanephroi cultured in ethanol for 5 days had reduced ureteric budding (Gray et al., 2010).

#### Inhibition of RAS Activity During Development

The role of the RAS, including its (pro)renin receptor, in renal development has been described above. It is not surprising therefore, that it was reported some time ago that oligohydramnios due to fetal anuria occurred when drugs that block the activity of the RAS were used to treat maternal hypertension (Lumbers et al., 1993; Barr, 1994). Earlier in renal development their administration causes renal papillary necrosis, lack of tubular development and hydronephrosis (Lasaitiene et al., 2003). It is important to appreciate these deleterious effects of RAS blockade on renal development because these drugs continue to be used to treat maternal hypertension. Nadeem et al. (2015) found that exposure even in the first trimester resulted in the early requirement for renal replacement therapy (RRT). They recommended that women who are on RAS blocking drugs and who are sexually active should have pregnancy testing if not using contraception (Cragan et al., 2015; Nadeem et al., 2015).

Conditional knockout of the (pro)renin receptor also causes abnormal renal development (Song and Yosypiv, 2011). The (pro)renin receptor not only activates prorenin, it also stimulates a number of signaling pathways including Wnt/β-catenin, ERK1/2 and PLZF pathways. These effects of (P)RR are independent from Ang II and are likely mediated by the receptor’s function as part of the vacuolar ATPase. Therefore, drugs that inhibit vacuolar ATPase may also affect renal development. These include bafilomycin and proton pump inhibitors (PPIs). PPIs have been suggested as a potential treatment for preeclampsia (Hastie et al., 2019) and have been used to treat pregnant women. They seem safe but it is also possible that they have subtle effects on fetal kidney development that do not manifest clinically for years.

#### Maternal Glucocorticoids

A Cochrane review Roberts et al. (2017) found that administration of corticosteroids to women at risk of preterm birth reduced neonatal mortality because they advanced maturation of the lung so improving postnatal respiratory function. Corticosteroids are also administered in pregnancy to treat a number of conditions (e.g., asthma and autoimmune disease). Glucocorticoids can, however, have unwanted effects on blood pressure and kidney function of offspring. Dodic et al. (1998) showed that a 48h infusion of dexamethasone (DEX) into pregnant ewes at 26–28 days of gestation (term 150 days) resulted in lambs that had higher blood pressures after birth compared with control animals (Dodic et al., 1998) and fewer bigger nephrons (Wintour et al., 2003). To see if tubuloglomerular feedback (TGF) was involved in the development of hypertension in young sheep whose mothers were treated with DEX at 26–27 days gestation, TGF was measured in both DEX treated fetuses and lambs. There was an increase in TGF sensitivity that persisted after birth and therefore could play a role in development of hypertension later in life (Turner et al., 2017). Since this difference in TGF sensitivity was no longer seen after inhibition of nitric oxide production it was concluded that there was lower tonic production of NO, which attenuates TGF sensitivity (Turner et al., 2017). The significance of this work is that similar changes in TGF sensitivity are seen in spontaneously hypertensive strains of rats [Milan and SHR (Persson et al., 1985; Boberg and Persson, 1986; Brannstrom and Arendshorst, 1999)]. It is worth noting that this change in TGF sensitivity occurred before there was any change in blood pressure.

Ortiz et al. (2001) showed in rats, that maternal dexamethasone (0.2 mg) given on days 15–16 and 17–18 of gestation reduced glomerular numbers of 2 months old male and female offspring. They later showed that only male offspring were hypertensive at 6 months of age (Ortiz et al., 2003). Both male and female offspring had significant glomerulosclerosis, although it was more severe in males. This work shows that a number of factors influence kidney development and the future health of offspring whose mothers were treated with synthetic steroids on the development of the kidney, including time of administration, sex and effects on blood pressure.

Significantly, Moritz et al. (2011) showed that naturally occurring steroids had effects similar to those of synthetic steroids on blood pressure (although the mechanisms underlying hypertension were different) and on the kidney. That is, naturally occurring steroids administered to the mother reduced glomerular number, altered the activity of the renal renin-angiotensin system and increased the expression of the epithelial sodium channel (α, β, and γ subunit) and the Na-K-ATPase (Moritz et al., 2011). This work is significant as it implies that anything resulting in severe maternal stress, which elevates maternal cortisol levels, could affect renal development.

#### Maternal Renal Insufficiency

It is known that renal disease in pregnant women is associated with adverse pregnancy outcomes (Blom et al., 2017) but does mild maternal renal insufficiency alter the development of the kidneys of her offspring?

The effects of mild maternal renal insufficiency on fetal renal function was studied in ewes who had one kidney removed and a branch of the renal artery in the remaining kidney occluded (subtotal nephrectomy; STNx) so that 30–50% of the remaining kidney became ischaemic (Gibson et al., 2006) before they were mated about 2–6 months after surgery. Ewes and fetuses were studied in the last fifth of gestation. The ewes had a raised serum creatinine and maternal GFR/kg bodyweight was only 54% that of intact pregnant ewes (Intact) (Gibson et al., 2006). Fetal GFR of STNx ewes was higher (about 1 ml/min) than the GFR of fetuses from Intact ewes. Both total and proximal tubular sodium reabsorption were reduced in fetuses of STNx ewes (Gibson et al., 2007). Moreover, infusion of amino acids known to stimulate ovine fetal RBF and GFR (Boyce et al., 2005) did not increase either RBF or GFR in fetuses of STNx ewes although it did cause an increase in both parameters in Intact fetuses (Gibson et al., 2007). Two weeks after birth, lambs from STNx (STNxL) and Intact (IntactL) ewes had a similar number of nephrons and their kidney weights were similar. However, STNxL had glomerular hypertrophy (glomeruli were about 30% larger) and there was a positive relationship between glomerular volume and urinary protein excretion. Therefore, exposure of the fetus to maternal renal insufficiency caused long-term changes in glomerular morphology (Brandon et al., 2008).

STNxL also had higher blood pressures at 26–27 days of age and there was abnormal programming of their RASs, in that the increase plasma renin levels in response to acute hemorrhage (1.6 ml/min/kg for 10 min) was attenuated (Brandon et al., 2011). Nor was the RAS in female STNxL as sensitive to altered salt intake as in IntactL (Brandon et al., 2009, 2011). In these 6-month old STNxL females, GFR was related to blood pressure and their plasma renin levels did not decrease, although plasma aldosterone levels did in response to a high salt intake. These animals had increased plasma sodium levels and reduced hematocrits; thus, they were volume expanded, probably because Ang II production was sustained in the presence of a high salt diet (Brandon et al., 2009).

To find out if these changes were related to dysregulation of tubuloglomerular feedback (TGF), studies were carried out in STNxF (Turner et al., 2015). In fact TGF was not different between STNxF and IntactF and it was concluded that the increase in GFR in STNx was related to an increase in filtration coefficient (Kf) (Turner et al., 2015). This fits with the glomerular hypertrophy described previously in STNxL. This finding is in contrast to the programming of TGF by maternal dexamethasone treatment given to the ewe in early gestation, which reduces glomerular number and causes hypertension in offspring (described above).

In conclusion, maternal renal dysfunction so mild that it does not interfere with normal fertility has profound effects on offspring kidney development, with glomerulomegaly and hyperfiltration predisposing to glomerulosclerosis in adult life and a blunted renin response to a high salt intake also contributing glomerulosclerosis and possibly causing hypertension.

## Prematurity

In the human, nephrogenesis is not complete until about 36 weeks of gestation. Therefore, the more premature the infant the greater the impact of prematurity on nephrogenesis.

Studies have shown that prematurely born infants have smaller kidneys (Kandasamy et al., 2013) and in premature infants total kidney volume almost doubled from 28 to 37 weeks, although they were still smaller than kidney weights of term neonates. Since the eGFRs were similar, prematurely born infants must have higher SNGFRs, which suggests that they are already hyperfiltering (Kandasamy et al., 2018). Sutherland et al. (2011) showed that glomerulogenesis was accelerated in preterm human infants after birth; these glomeruli were also larger and there was a significantly greater percentage of abnormal glomeruli. Li et al. (2020) using ultrasound, came to a similar conclusion; the renal cortical region underwent accelerated growth after birth, but postnatal growth of the medulla was retarded. In the premature baboon (independent of steroid exposure) there was a high proportion of abnormal glomeruli (ranging from 0.2 to 18%) (Gubhaju et al., 2009).

The postnatal environment for the preterm infant is an environment in which hypoxia, hypotension, and infection are likely and antibiotics and other drugs that may be nephrotoxic are used. Thus, there are potentially a number of causes of disrupted kidney development in the preterm infant and its impact on adult renal function and cardiovascular health is largely unknown.

Keijzer-Veen et al. (2007) assessed renal function and kidney size in a cohort of 29 young adults (20 years old) who were born preterm (<32 weeks gestation) (Keijzer-Veen et al., 2007) and did not find any difference in renal function or kidney size between the preterm and control groups. However, a more recent study by South et al. (2019) published follow up data from a cohort of 96 adolescents (14 years) who were born preterm (<28 weeks) and showed that these children had elevated blood pressures and lower eGFRs compared with their peers were who were born at term (South et al., 2019).

## Transgenerational Modification of Renal Development and Renal Disease

A multiplicity of factors account for the high prevalence of CKD/ESRD in adult Indigenous Australians (Hoy et al., 1998b) but an underlying factor is oligonephropathy associated with low birth weight. Hoy et al. (1998a) first showed an association in Indigenous Australians between low birth weight and renal disease and in particular between low birth weight and a high albumin/creatinine ratio. Indigenous Australians have fewer nephrons; i.e., there are about 200,000 fewer nephrons or only 75% of those found in non-Indigenous Australians (Hoy et al., 2006). This means that there is probably pre-existing glomerular hyperfiltration and, since both obesity and diabetes mellitus also cause glomerular hyperfiltration (see above), they, together with the lower nephron number accelerate progression to CKD.

In Indigenous Australians the prevalence of ESRD and RRT is 6–8 times non-Indigenous Australians (age adjusted) and the median age at which they developed ESRD is about 30 years younger than non-Indigenous Australians. It is highly likely that many Indigenous Australian women have mild renal dysfunction (Hughes et al., 2018; Hoy et al., 2020) as evidenced by albuminuria, which is a robust measure of risk of future development of renal and cardiovascular disease (Hoy and McDonald, 2004).

Pringle et al. (2018) have shown that urinary protein/creatinine ratios were higher in Indigenous pregnant women than in non-Indigenous pregnant women, all of whom had uncomplicated pregnancy outcomes (Pringle et al., 2018). Indigenous Australians also have a high prevalence of small for gestational age babies and premature birth (Gingery et al., 2009) and an increased incidence of obesity and diabetes mellitus (Pringle et al., 2019). Kandasamy et al. (2014) found that Indigenous Australian infants had smaller kidney volumes than non-Indigenous infants. Since there was no difference in GFR (expressed ml.min.1.73 m^2^), it follows that single nephron GFR (SNGFR) must have been increased in Indigenous neonates.

While there are genetic polymorphisms that contribute to this epidemic of CKD (Duffy et al., 2016), some of those SNPs commonly associated with hypertension and CKD in other populations have a very low prevalence in Indigenous Central Australians (Grimson et al., 2016). Conversely, the prevalence of the DD genotype in the ACE gene is higher in Indigenous Australians requiring dialysis than it is in healthy controls, suggesting that overactivity of the RAS exacerbates chronic renal disease (Lester et al., 1999).

The impact of the sheep data showing the impact of maternal subtotal nephrectomy prior to conception on renal function of offspring both prenatally and postnatally clearly demonstrates that maternal renal health is a determinant of offspring renal health. That is, there is a transgenerational effect on renal health as well as a genetic contribution Duffy et al. (2016) the ACE I/D polymorphism and p53 polymorphisms account for 15% of the heritability of a high albumin/creatinine and blood pressure.

## Conclusion

A lower nephron number and larger glomerular volume as a result of hyperfiltration is therefore the transgenerational substrate for the continuing high risk of ESRD and RRT, particularly in Indigenous communities. However, throughout life, a progressive reduction in nephron number due to nephrosclerosis occurs (Figure 3). Thus, a low nephron number at birth, the imposition of obesity, diabetes mellitus and (to a lesser extent these days) post-streptococcal glomerulonephritis accelerates this glomerular loss so CKD occurs at a younger age. Since nephron number has been determined by the time of birth, considerable attention should be given to those maternal lifestyle factors that affect nephrogenesis (such as alcohol, smoking, obesity, adequate nutrition, and micronutrients). However, the effects of maternal renal health on the risk of early onset of CKD in their offspring will only be eliminated when the prevalence of renal dysfunction in pregnant women is reduced. As emphasized at the beginning of this review, CKD (as measured by albumin/creatinine) is a significant a risk factor for hypertension and cardiovascular disease. Therefore, any progress in reducing the prevalence of CKD also brings further benefit by reducing cardiovascular disease.

We recommend that kidney volumes should be measured at birth and infants with small kidney volumes or those who are premature be monitored throughout life by measuring eGFR and albumin/creatinine ratio. Particular attention should be paid to their level of nutrition and programs that prevent obesity and type II diabetes mellitus introduced into the routine delivery of primary health care. Hypertension requires treatment and continuous surveillance. Finally, it is tempting to suggest that ingestion of large amounts of protein should be advised against.

## Author Contributions

EL wrote the first draft of the manuscript. KP, YK, SD, AB, and KG contributed to manuscript revision. All authors approved the final submission.

## Conflict of Interest

The authors declare that the research was conducted in the absence of any commercial or financial relationships that could be construed as a potential conflict of interest.
